# Optimizing CAD/CAM ceramics: a comparative study of polishing and glazing effects on surface quality and aesthetic properties following coffee thermocycling

**DOI:** 10.1186/s12903-026-08142-8

**Published:** 2026-03-21

**Authors:** Sena Kamacı Ergül, Diler Deniz, Cumhur Altıntaş

**Affiliations:** 1 Turkish Ministry of Health, Mamak Oral and Dental Health Center, Ankara, Turkey; 2 Department of Prosthodontics, Faculty of Dentistry, Hacettepe University, Ankara, 06100 Turkey

**Keywords:** Zirconia, Ceramic, Roughness, Colour, Translucency, Glaze, Polish

## Abstract

**Background:**

This in vitro study aims to evaluate the effects of two different surface finishing procedures-glazing and polishing-on the stability of colour, the translucency, and the roughness of surface with three CAD/CAM ceramic materials: lithium disilicate ceramic (LDC), zirconia-reinforced lithium silicate ceramic (ZLC), and translucent zirconia (TrZ). The process is evaluated before and after coffee thermocycling.

**Method:**

A total of 120 samples (1.0 ± 0.1 mm) were prepared from LDC (IPS e.max CAD HT A2), ZLC (Celtra Duo HT A2), and TrZ (Cercon HT A2). All samples were subjected to standardized grinding for 20 s to simulate chairside adjustments and were then assigned to glaze (G) or polish (P) subgroups (*n* = 20). Surface roughness (Ra, Rz) was measured by using a contact profilometer, while colour coordinates (L*, a*, b*), colour difference (ΔE₀₀) (∆E00), and translucency parameter (TP) were recorded with a colorimeter. All samples underwent 10,000 coffee thermocycles (5–55 °C). Data were analysed by using one-way ANOVA, repeated-measures ANOVA, Kruskal–Wallis, and appropriate post-hoc tests (α = 0.05).

**Result:**

It is seen that grinding produced the highest Ra and Rz values in ZLC, followed by LDC and TrZ. When surface treatments were completed, polished TrZ showed a significantly lower Ra compared with glazed TrZ, while ZLC and LDC exhibited lower roughness after glazing, although not significantly. Only the ZLC-P group demonstrated a perceptible colour change, yet all groups remained within the clinically acceptable threshold (< 2.23). Before and after thermocycling, LDC showed the highest TP values, followed by ZLC and TrZ. Thermocycling significantly increased Ra (except in TrZ-P and ZLC-P) and decreased TP values in all materials except LDC-P.

**Conclusion:**

Based on the results, it is concluded that surface finishing techniques and coffee thermocycling affect the optical and surface properties of CAD/CAM ceramics. Glazing generally provides smoother surfaces for glass-ceramics, whereas polishing has superior outcomes for translucent zirconia.

## Background

The demand for highly aesthetic restorations has increased substantially in recent years and supported by rapid developments in digital dentistry. Although metal-ceramic restorations are considered as reliable treatment alternatives, their well-known drawbacks such as the shadowing of the metal substructure, potential allergic reactions, veneer porcelain chipping, and insufficient optical mimicry of natural dentition have triggered the search for new restorative systems [1–3]. Consequently, biocompatible full ceramic materials with superior mechanical and aesthetic properties have been introduced and are now widely implemented in clinical practice [1–2].

Yttria-stabilized tetragonal zirconia polycrystal (Y-TZP) has emerged as a strong alternative to metal frameworks due to its high mechanical strength; however, its inherent opacity requires veneering with feldspathic porcelain, which poses a significant risk of chipping [3–5]. This challenge has accelerated the development of monolithic ceramics because they do not require a veneering layer and therefore eliminate veneer related failures. Recent advancements have led to the introduction of more translucent zirconia systems, as well as lithium disilicate (LDC) and zirconia-reinforced lithium silicate (ZLC) ceramics, the latter containing approximately 10% zirconia for enhanced mechanical performance [6–8]. These materials provide an improved balance between aesthetics and strength, facilitating their widespread use in anterior and posterior restorations [9].

Although CAD-CAM technology enables fast and precise fabrication of monolithic restorations, intraoral adjustment is frequently required. Such adjustments generate surface irregularities that might negatively affect biological compatibility, optical behaviour, and mechanical performance [10]. Surface finishing protocols namely glazing and mechanical polishing are used to correct these defects; however, the literature still presents limited and inconsistent data regarding their effects on roughness (Ra-Arithmetic mean surface roughness, Rz- Mean peak-to-valley height), colour stability (∆E), and translucency (TP), particularly following thermal aging procedures.

Thermal aging is an important factor that can influence the long-term performance of CAD-CAM ceramic restorations, as repeated temperature fluctuations in the oral environment might induce microstructural changes, phase transitions, and surface alterations within these materials. Such changes can affect optical behaviour, particularly colour stability and translucency, which are essential for maintaining the natural appearance of monolithic ceramics. Colour shifts might occur due to increased surface roughness, pigment penetration, or chemical changes within the glassy matrix, while reductions in translucency can diminish the material’s ability to mimic the depth and light scattering characteristics of natural enamel. Recent investigations have indicated that thermal cycling and exposure to staining agents can modify the optical properties of lithium based and zirconia-based ceramics to varying degrees, highlighting the need to better understand how different surface finishing approaches interact with aging processes to influence clinical outcomes [11–12].

Surface quality plays a crucial role in the long-term success of ceramic restorations. A well-polished surface resists biofilm accumulation, reduces plaque retention, and contributes to periodontal health. Notably, patients can detect roughness changes smaller than 1 μm with tongue proprioception, and values above are associated with increased staining and bacterial adhesion [13]. Moreover, smooth surfaces decrease stress concentrations that might act as crack initiation sites, thereby improving fracture resistance. Polished ceramics also minimize antagonist wear, prevent loss of occlusal stability, reduce corrosion risks, and enhance light reflection for improved aesthetic outcomes [14].

Although CAD/CAM ceramic materials are widely used in contemporary prosthodontics, their long-term aesthetic performance is influenced by multiple clinical variables, particularly surface finishing procedures and exposure to aging conditions. Previous studies have generally evaluated material type, finishing protocols, or thermocycling procedures independently, and multifactorial data addressing their combined effects remain limited [15–17]. In clinical practice, restorations are subjected to surface adjustments followed by repeated thermal challenges, suggesting that these variables may interact rather than act in isolation. In this multifactorial experimental design, the null hypothesis of this study was that (1) the type of CAD/CAM ceramic material, (2) the surface finishing protocol (polishing versus glazing), and (3) the artificial aging procedure (coffee thermocycling) would have no statistically significant effect on surface roughness, color stability (∆E), or translucency parameter (TP).

## Method

### Sample preparation

**Fig. 1:**
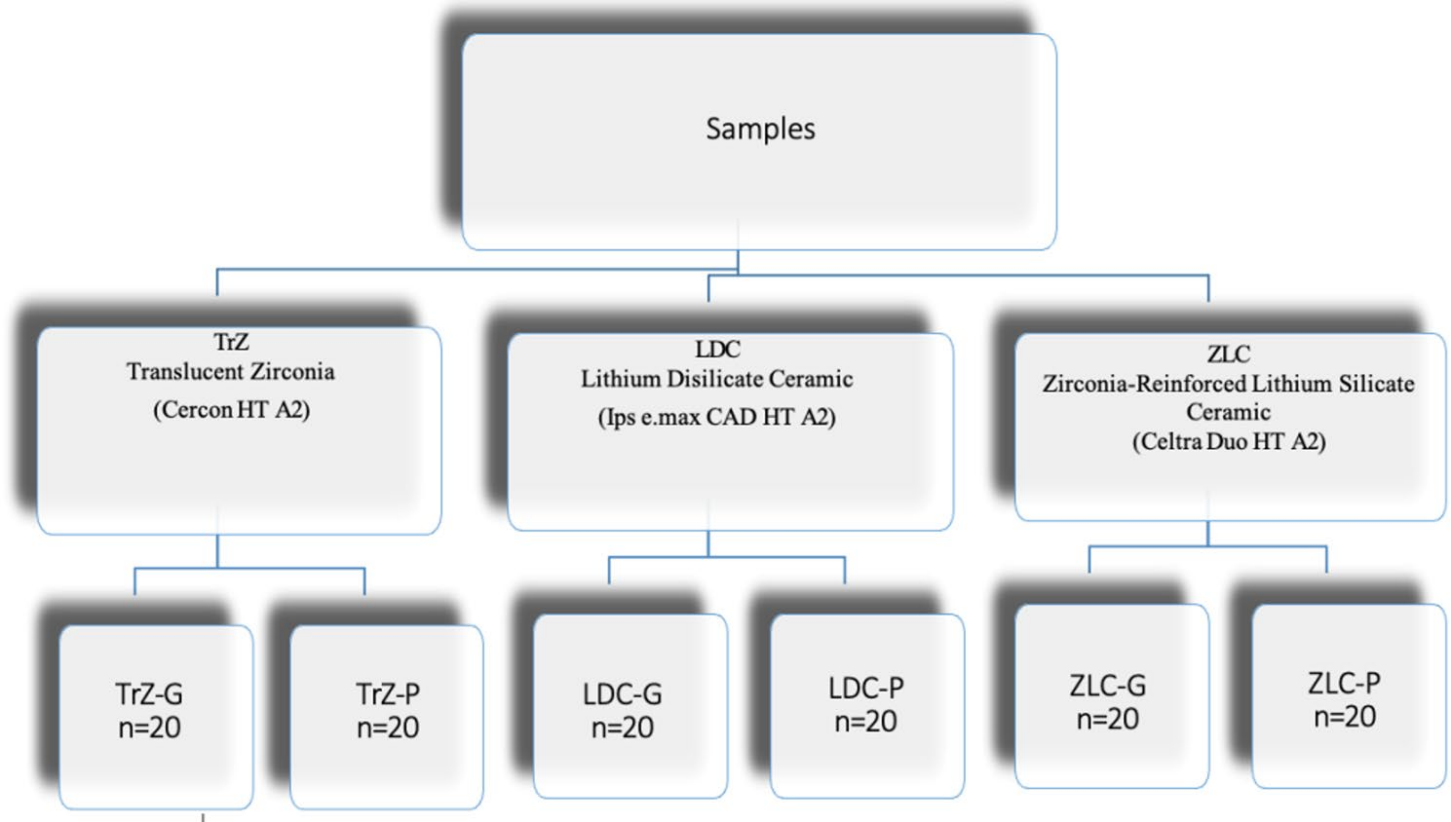
Schematic representation of the study groups. TrZ-G: Glazed translucent zirconia samples. TrZ-P: Polished translucent zirconia samples. LDC-G: Glazed lithium disilicate ceramic samples. LDC-P: Polished lithium disilicate ceramic samples. ZLC-G: Glazed zirconia reinforced silicate ceramic samples. ZLC-P: Polished zirconia reinforced silicate ceramic samples

Based on G*Power (v3.1) calculations and effect sizes reported in comparable studies, a sample size of 20 specimens per subgroup has been calculated to provide a statistical power of at least 0.80 (α = 0.05). A total of 120 samples have been fabricated (*n* = 40 for each material), and the final thickness of all samples is standardized at 1.0 ± 0.1 mm. The materials used in this study includes translucent zirconia-TrZ (Cercon HT A2; DeguDent GmbH, Hanau-Wolfgang, Germany), lithium disilicate glass-cermaic-LDC (IPS e.max CAD HT A2; Ivoclar Vivadent, Schaan, Liechtenstein), and zirconia-reinforced lithium silicate ceramic-ZLC (Celtra Duo HT A2; DeguDent GmbH). Each material group is further divided into glazing and polishing subgroups (TrZ-G/TrZ-P, ZLC-G/ZLC-P, LDC-G/LDC-P), with 20 samples allocated to each condition (Fig. 1).

LDC samples are obtained by sectioning IPS e.max CAD HT A2 blocks with a precision cutting device (Metkon Microcut 201, Metkon Endüstriyel, Bursa, Türkiye) at an initial thickness of 1.1 mm. Their surfaces are subsequently smoothed for 30 s using 800, 1000, and 1200 grit silicon carbide abrasives on a grinding unit (Gripo 2 V). Crystallization is conducted in a Programat P500 furnace (Ivoclar Vivadent) following the manufacturer’s recommended firing protocol. Presintered TrZ blocks (Cercon HT A2) are trimmed into 14 × 14 × 1.35 mm pieces by using the same cutting device, taking sintering shrinkage into account, as well. The final sintering is performed at 1500 °C for 5.5 h in a TABEO-1/S/ZIRKON-100 furnace (MIHM-VOGT GmbH). Similar to the LDC samples, the surfaces are also refined for 30 s with progressively finer abrasive papers under water cooling. ZLC samples (Celtra Duo HT A2) are also sectioned to 1.1 mm thickness and polished by using the same abrasive sequence. The final thickness of all samples across the three material categories has been verified by using a digital calliper to ensure consistency at 1.0 ± 0.1 mm. For all three materials, the mentioned procedures are carried out in accordance with the respective manufacturers’ protocol.

All samples are subjected to a controlled grinding performed by a single operator in order to ensure standardized surface conditions before polishing or glazing. A laboratory handpiece (KaVo EWL 4990; KaVo Dental GmbH) connected to a micromotor (KaVo EWL K11) is used with a red-band diamond bur operated at 20,000 rpm for 20 s in a unidirectional motion. A new bur is introduced after every 10 samples. Following this procedure, samples from each material are randomly assigned to the glazing or polishing subgroups.

For LDC samples, IPS Ivocolor Glaze (Ivoclar Vivadent) is mixed according to the recommended manufacturer protocol, applied as a uniform single coat, and fired in a Programat P500 furnace following the prescribed glazing cycle. For ZLC samples, Celtra Universal Overglaze (DeguDent) is applied in paste form and fired in a Multimat Touch&Press furnace (Dentsply GmbH) in accordance with the manufacturer’s glazing protocol. For TrZ samples Dentsply Sirona Universal Overglaze (DeguDent) is similarly applied in a single layer and fired in the same furnace.

Polishing procedures differs according to material. For LDC, the three-step Optrafine polishing system (Ivoclar Vivadent) is used. Under continuous water spray, finishing rubbers (light blue) are applied at 12,000 rpm for 60 s, followed by polishing rubbers (dark blue) for an additional 60 s. High-gloss polishing is achieved by applying polishing paste using nylon brushes at 7,000 rpm for 60 s. For ZLC, the two-step StarTec system (Edenta AG) is applied. The medium-grit purple wheel is used at 10,000 rpm for 60 s, followed by the ultra-fine yellow wheel at 7,000 rpm for 60 s, both under water cooling. For TrZ, the EVE Diacera system (EVE Ernst Vetter GmbH) is used. Medium-grit green wheels and high-gloss orange wheels are applied sequentially for 60 s at the speeds recommended by the manufacturer.

All polishing and glazing procedures are carried out by the same operator. After treatment, all samples are ultrasonically cleaned in deionized water for 10 min (Biosonic JR, Whaledent Int., NY, USA).

A comprehensive list of all materials used in the study is provided in Table 1.

**Table 1:** Materials used in the study

**Material**	**Brand**	**Manufacturer**	
Translucent Zirconia (TrZ)	Cercon HT A2	DeguDent GmbH Hanau- Wolfgang, Germany	LOT18032409
Lithium Disilicate Ceramic (LDC)	Ips e.max CAD HT A2	Ivoclar Vivadent, Schaan, Liechtenstein	LOTX39494
Zirconia-Reinforced Lithium Silicate Ceramic (ZLC)	Celtra Duo HT A2	DeguDent GmbH Hanau- Wolfgang, Germany	LOT16000495
Glaze (for TrZ)	Dentsply Sirona Universal Overglaze	DeguDent GmbH Hanau- Wolfgang, Germany	Not available
Glaze (for LDC)	IPS Ivocolor Glaze	Ivoclar Vivadent, Schaan, Liechtenstein	Not available
Glaze (for ZLC)	Celtra Universal Overglaze	DeguDent GmbH Hanau- Wolfgang, Germany	Not available
Polishing Kit (for TrZ)	EVE Diacera Polishing kit	EVA Ernst Vetter GmbH, Pforzheim, Germany	LOT406142
Polishing Kit (for LDC)	Optrafine Polishing Kit	Ivoclar Vivadent, Schaan, Liechtenstein	LOTXL0691
Polishing Kit (for ZLC)	StarTec Polishing Kit	Edenta AG, Hauptstrasse, Switzerland	Green-LOTB05.001, Yellow-LOTF03.001
Coffee Solution	Nescafe Classic	Nestlé S.A. Vevey, Switzerland	Not available

#### Surface roughness measurements

The measurements related to the surface roughness is conducted by using a contact profilometer (Perthometer M2, Mahr GmbH, Germany) equipped with a 2-µm stylus tip angle of 60° and a 100-µm measuring range. The cut-off length is set at 0.8 mm, with a total measurement length of 5.6 mm. For each sample, three parallel measurements are taken from the central region both before and after thermocycling, and the mean Ra and Rz values are recorded.

#### Colour and Translucency Measurements

The assessments related to the colour and translucency are performed by using a tristimulus colorimeter (Konica Minolta CR-321, Tokyo, Japan), calibrated before each series by using the manufacturer’s standard white calibration plate. Measurements are done inside a controlled illumination cabinet designed to simulate daylight conditions.

Each sample is evaluated on black, white, and grey backgrounds. For each background, three readings are taken from the central area of the sample, and the average L*, a*, and b* values are recorded. The colour differences (ΔE₀₀) are calculated using the CIEDE2000 formula from the grey-background measurements obtained before and after thermocycling.

$$\begin{aligned} &\:{\Delta\:}{E}_{\left\{00\right\}\left(L{*}_{1},\:a{*}_{1},\:b{*}_{1};\:L{*}_{2},\:a{*}_{2},\:b{*}_{2}\right)}=\\&\:\sqrt{\left\{{\left(\frac{{\Delta\:}{L}^{{\prime\:}}}{{k}_{L}{S}_{L}}\right)}^{2}+\:{\left(\frac{{\Delta\:}{C}^{{\prime\:}}}{{k}_{C}{S}_{C}}\right)}^{2}+\:{\left(\frac{{\Delta\:}{H}^{{\prime\:}}}{{k}_{H}{S}_{H}}\right)}^{2}+\:{R}_{T}\left(\frac{{\Delta\:}{C}^{{\prime\:}}}{{k}_{C}{S}_{C}}\right)\left(\frac{{\Delta\:}{H}^{{\prime\:}}}{{k}_{H}{S}_{H}}\right)\right\}} \end{aligned}$$.

The translucency parameter (TP) is determined using the L*, a*, and b* values measured over black and white backgrounds according to:

$$\:TP=\sqrt{\left\{{\left({L}_{W}-{L}_{B}\right)}^{2}+{\left({a}_{W}-{a}_{B}\right)}^{2}+{\left({b}_{W}-{b}_{B}\right)}^{2}\right\}}$$

(W refers to the white background and B to the black background)

In this study, thermal aging is conducted using a thermocycling device (SD Mechatronic Thermocycler, Seelbach, Germany). A coffee solution prepared from soluble coffee (Nescafe Classic, 1 g/100 mL, Nestlé S.A., Vevey, Switzerland) served as the staining medium and is used in both the hot and cold baths. The samples underwent 10,000 cycles between 5 °C and 55 °C, with a dwell time of 10 s in each bath and a transfer interval of 5 s. This thermocycling protocol has been widely used to simulate oral aging conditions and evaluate optical and mechanical properties of dental materials [18]. Following the thermal aging procedure, the surface roughness, the colour, and the translucency have been assessed and calculated using the same methods described for the pre-aging measurements. All statistical analyses are performed by using IBM SPSS Statistics 22.0 (IBM Corp., Armonk, NY, USA). The normality of continuous variables has been evaluated by using the Shapiro–Wilk test. Statistical tests are selected according to data distribution and study design. Non-normally distributed ΔE₀₀ data have been analysed using the Kruskal–Wallis test, followed by Dunnett’s post-hoc test when appropriate. Translucency parameter (TP) values have been analysed by using repeated-measures ANOVA, with Duncan’s post-hoc procedure applied to allow detailed subgroup comparisons among ceramic materials and surface finishing protocols. Changes in TP before and after thermocycling are assessed via paired t-tests. Initial surface roughness values after grinding are compared using one-way ANOVA, whereas pre- and post-thermocycling roughness data have been evaluated using repeated-measures ANOVA followed by Duncan’s post-hoc test. Statistical findings are cautiously interpreted by considering descriptive statistical outcomes together with clinical relevance. Statistical significance is set at *p* < 0.05.

## Result

### Surface roughness after controlled grinding

Following the standardized grinding procedure, both Ra and Rz values differed significantly among the three CAD-CAM ceramic materials (*p* < 0.05). ZLC exhibited the highest surface roughness (Ra: 2.59 ± 0.29 μm; Rz: 16.11 ± 1.48 μm), whereas TrZ demonstrated the lowest values (Ra: 1.08 ± 0.17 μm; Rz: 7.26 ± 0.84 μm) **(**Table 2). Duncan’s multiple comparison test has confirmed statistically significant differences among all materials for both parameters (Table 3).

**Table 2:** Descriptive statistics and one-way ANOVA results for Ra and Rz after controlled grinding

Descriptive Statistics									
Parameter	Material	N	Mean	SD	SE	95% CI (Lower–Upper)		Min	Max
Ra (µm)	ZLC	40	2.59	0.29	0.045	2.50 – 2.68		2.09	3.39
	TrZ	40	1.08	0.17	0.018	1.04 – 1.12		0.84	1.40
	LDC	40	2.00	0.30	0.048	1.91 – 2.10		1.58	2.63
Rz (µm)	ZLC	40	16.11	1.48	0.235	15.64 – 16.59		13.13	19.97
	TrZ	40	7.26	0.84	0.133	6.99 – 7.53		5.41	9.85
	LDC	40	12.30	1.96	0.310	11.67 – 12.93		9.43	16.40

One-way ANOVA Results									
Parameter	Source		SS	df		MS	F	p	
Ra	Between Groups		46.494	2		23.247	375.929	0.000*	
Ra	Within Groups		7.235	117		0.062			
Ra	Total		53.729	119					
Rz	Between Groups		1575.808	2		787.904	349.947	0.000*	
Rz	Within Groups		263.425	117		2.251			
Rz	Total		1839.233	119					

**Table 3:** Duncan post-hoc comparison results for Ra and Rz after abrasion

Material	Mean Ra (µm)	Ra Subset	Mean Rz (µm)	Rz Subset
TrZ	1.08	A	7.26	A
LDC	2.00	B	12.30	B
ZLC	2.59	C	16.11	C

*TrZ *Translucent zirconia, *LDC *Lithium disilicate ceramic, *ZLC *Zirconia-reinforced lithium silicate ceramic, *R*a Arithmetic mean surface roughness, *Rz *Mean peak-to-valley height. Groups sharing the same letter are not significantly different (*p* < 0.05)

#### Pre-thermocycling surface roughness after finishing procedures

After completing the procedures, surface roughness values varied depending on the material type and the surface treatment applied. For Ra, the lowest values have been recorded in the ZLC-G and TrZ-P groups, while the highest values are observed in LDC-P and TrZ-G. For Rz, glaze groups showed a markedly lower roughness than their corresponding polishing groups across all materials.

#### Changes following thermocycling

Repeated-measures ANOVA have revealed a significant effect of thermocycling on surface roughness parameters. Ra values increased significantly after thermocycling (*p* = 0.025), except in the TrZ-P and ZLC-P groups, which showed no meaningful change. In contrast, Rz values were not significantly affected by thermocycling (*p* = 0.103). Furthermore, no significant group × time interaction was observed for either Ra or Rz, indicating that the effect of thermocycling has been consistent across materials and surface treatments.

Post-thermocycling multiple comparisons revealed significant differences among the six experimental subgroups for both Ra and Rz (*p* < 0.05). Overall, glazed specimens exhibited lower surface roughness than polished specimens in both the ZLC and LDC groups, whereas TrZ specimens showed only minimal differences between glazing and polishing after thermocycling **(**Tables 4 and 5).

**Table 4:** Ra and Rz values before and after thermocycling for glaze and polishing groups

Material	Surface Treatment	Ra Before	Ra After	Rz Before	Rz After
TrZ	Glaze	0.52 (0.24)	0.59 (0.37)	2.52 (1.16)	2.50 (1.63)
TrZ	Polish	0.36 (0.15)	0.36 (0.12)	2.54 (1.46)	2.65 (1.17)
LDC	Glaze	0.40 (0.20)	0.55 (0.22)	1.60 (0.72)	2.25 (1.00)
LDC	Polish	0.52 (0.13)	0.57 (0.16)	3.21 (1.00)	3.74 (1.26)
ZLC	Glaze	0.30 (0.20)	0.36 (0.26)	1.32 (0.72)	1.43 (1.10)
ZLC	Polish	0.42 (0.16)	0.42 (0.16)	3.71 (1.35)	3.83 (1.64)

*TrZ *Translucent zirconia, *LDC *Lithium disilicate ceramic, *ZLC *Zirconia-reinforced lithium silicate ceramic, *Ra *Arithmetic mean surface roughness, *Rz  *Mean peak-to-valley height, *SD *Standard deviation

**Table 5:** Duncan post-hoc comparison results for Ra and Rz after surface finishing and thermocycling

Material – Surface Treatment	Mean Ra (µm)	Ra Subset	Mean Rz (µm)	Rz Subset
TrZ – G	0.55	C	2.51	B
TrZ – P	0.36	A	2.59	B
LDC – G	0.47	B	1.93	A
LDC – P	0.54	C	3.48	C
ZLC – G	0.33	A	1.37	A
ZLC – P	0.42	B	3.77	C

*TrZ *Translucent zirconia, *LDC *Lithium disilicate ceramic, *ZLC *Zirconia-reinforced lithium silicate ceramic, *Ra *Arithmetic mean roughness, *Rz *Mean peak-to-valley height

Groups sharing the same letter are not significantly different (*p* < 0.05)

#### Colour difference

**Fig. 2:**
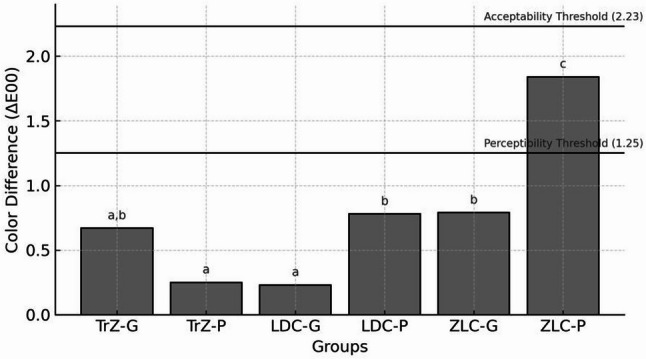
Color difference (ΔE₀₀) (ΔE00) values of the groups

Descriptive statistics for colour differences across all materials and surface treatments are presented in Table 6, with distribution patterns illustrated in Fig. 2. Overall, most groups exhibited ΔE values below the commonly accepted clinical threshold (ΔE00 = 2.23), indicating limited perceptible discoloration after thermocycling. The exception is the ZLC-P group, which showed markedly higher ΔE values exceeding the perceptibility threshold.

**Table 6:** The comparison of Colour differences (ΔE₀₀) Values According to Material and Surface Treatment

Surface Treatment	TrZ (Median, Min–Max)	LDC (Median, Min–Max)	ZLC (Median, Min–Max)	*p*-value
Glaze	0.67 (0.18–0.90) A, a	0.23 (0.13–0.94) B, a	0.79 (0.20–1.25) A, a	< 0.001
Polish	0.25 (0.14–0.80) B, a	0.78 (0.22–1.83) C, b	1.84 (1.16–2.10) A, b	< 0.001
p-value	0.061	0.001	0.002	

Values are presented as median (minimum–maximum). Different uppercase letters (A, B, C) indicate statistically significant differences among materials within the same surface treatment (*p* < 0.05). Different lowercase letters (a, b) indicate statistically significant differences between glaze and polish within the same material (*p* < 0.05). Statistical comparisons were performed using the Kruskal–Wallis test and Dunn’s multiple comparison test

When glaze was used as the constant finishing method, no statistically significant differences have been observed between TrZ and LDC (*p* = 0.069) or between TrZ and ZLC (*p* > 0.05). However, a significant difference is detected between the LDC and ZLC glaze subgroups (*p* = 0.003). When polishing was kept constant, all three materials differed significantly from each other (TrZ–LDC: *p* = 0.001; LDC–ZLC: *p* = 0.008; TrZ–ZLC: *p* < 0.001). Within-material comparisons have revealed no significant difference between glaze and polish in the TrZ groups (*p* = 0.61), whereas both LDC and ZLC have exhibited significantly greater discoloration in the polished subgroups compared with their glazed counterparts (LDC: *p* = 0.001; ZLC: *p* = 0.002).

Collectively, the findings indicate that polishing generally led to higher ΔE values than glazing, with ZLC being the most susceptible to colour change, particularly when polished. The lowest discoloration has been observed in the LDC-Glaze group, whereas the highest is recorded in the ZLC-P group.

Mean color difference (ΔE₀₀) (ΔE00) values of the six experimental groups after surface finishing and thermocycling: translucent zirconia glazed (TrZ-G), translucent zirconia polished (TrZ-P), lithium disilicate ceramic glazed (LDC-G), lithium disilicate ceramic. polished (LDC-P), zirconia-reinforced lithium silicate ceramic glazed (ZLC-G), and zirconia-reinforced lithium silicate ceramic polished (ZLC-P). The dashed horizontal lines represent the perceptibility threshold (ΔE00 = 1.25) and the acceptability threshold (ΔE00 = 2.23). Different lowercase letters above the bars indicate statistically significant differences among groups (*p* < 0.05). TrZ: translucent zirconia; LDC: lithium disilicate ceramic; ZLC: zirconia-reinforced lithium silicate.

#### Translucency (TP)

Thermocycling significantly affected the translucency parameter (TP) across materials and surface treatments (repeated-measures ANOVA, *p* < 0.05). A significant group × time interaction is observed (*p* < 0.05), prompting subgroup analyses. Before thermocycling, all materials exhibited statistically different TP values within the glaze subgroups (*p* < 0.05). ZLC and LDC showed comparable translucency under polishing (*p* > 0.05), whereas TrZ demonstrated significantly lower TP values than both materials regardless of the surface treatment (*p* < 0.05). Within each material, glaze and polishing procedures have produced significantly different TP values for ZLC, LDC, and TrZ (*p* < 0.05).

After thermocycling, differences among materials remained significant for both glaze and polishing subgroups (*p* < 0.05). TrZ consistently exhibited the lowest TP values, while LDC showed the highest translucency under both finishing conditions. Surface treatment continued to significantly influence TP within all materials (*p* < 0.05), with glaze generally yielding higher translucency than polishing for LDC and ZLC, whereas TrZ demonstrated reduced translucency under both treatments.

**Fig. 3:**
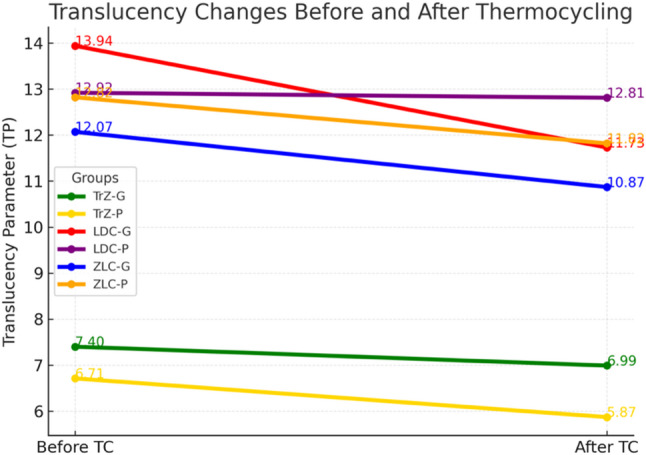
Translucency parameter (TP) changes before and after thermocycling for all experimental groups. TrZ: translucent zirconia; LDC: lithium disilicate ceramic; ZLC: zirconia-reinforced lithium silicate; G: glazing; P: polishing. Lines represent mean TP values before and after thermocycling

Paired-samples t-tests showed that thermocycling caused a significant decrease in TP for ZLC-G, ZLC-P, TrZ-G, and TrZ-P (*p* < 0.05), as well as for LDC-G (*p* < 0.05). In contrast, TP reduction in the LDC-P subgroup is not statistically significant (*p* = 0.563). Among all groups, only LDC-G exhibited a translucency change (ΔTP00 > 1.33) exceeding the perceptibility threshold, while all TP reductions remained below the clinical acceptability limit (ΔTP00 = 4.43). Overall, all groups have showed a decrease in translucency following thermocycling (Table 7, Fig. 3).

**Table 7:** TP Values and paired t-test results

Group	TP Before (Mean ± SD)	TP After (Mean ± SD)	Mean Difference	t-value	df	*p*-value
TrZ-G	7.40 ± 0.40	6.99 ± 0.73	0.42	2.16	19	0.044
TrZ-P	6.71 ± 0.51	5.87 ± 0.22	0.84	6.67	19	0.000
LDC-G	13.94 ± 0.36	11.73 ± 0.62	2.22	12.20	19	0.000
LDC-P	12.92 ± 1.32	12.81 ± 0.62	0.11	0.59	19	0.563
ZLC-G	12.07 ± 1.17	10.87 ± 1.57	1.20	4.35	19	0.000
ZLC-P	12.82 ± 0.89	11.82 ± 1.00	1.00	2.72	19	0.014

*TrZ *Translucent zirconia, *LDC *Lithium disilicate ceramic, *ZLC *Zirconia-reinforced lithium silicate, *G *Glazing, *P *Polishing. Statistical analysis performed using paired t-tests (*p* < 0.05 considered significant)

#### SEM analysis

**Fig. 4:**
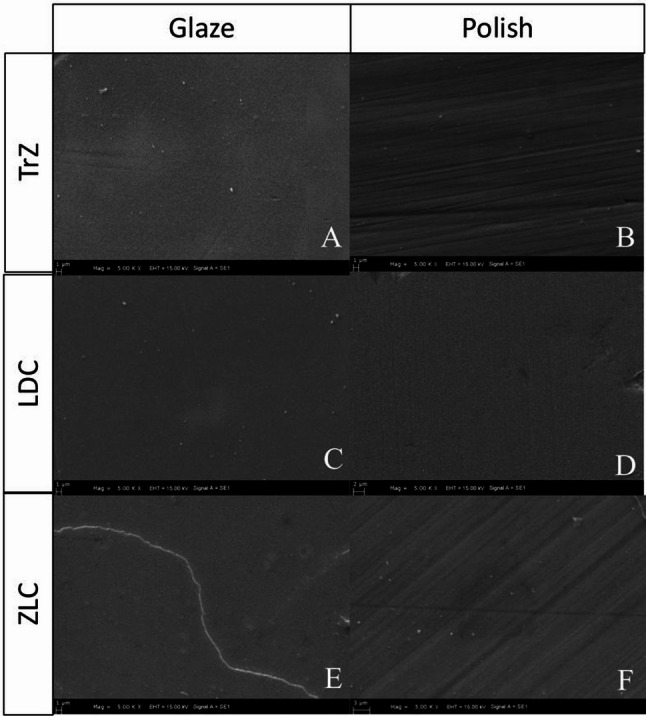
Surface morphology according to surface treatment. SEM images of ceramic specimens by group: (**A**) TrZ-G, (**B**) TrZ-P, (**C**) LDC-G, (**D**) LDC-P, (**E**) ZLC-G, and (**F**) ZLC-P. TrZ: translucent zirconia; LDC: lithium disilicate ceramic; ZLC: zirconia-reinforced lithium silicate

SEM evaluation has revealed that abrasion-induced scratches are markedly reduced in all polished groups, whereas glazed surfaces have exhibited smoother and more uniform morphologies. ZLC-G showed occasional glaze-layer irregularities, while ZLC-P displayed faint scratches with minor surface pits. LDC-G presented a homogenous surface, whereas LDC-P retained a few deep irregularities not fully eliminated by polishing. TrZ-G exhibited a smooth surface, while TrZ-P showed localized deep scratches. Overall, glazing has produced the smoothest surfaces, whereas the effectiveness of polishing has varied depending on the material (Fig. 4).

## Discussion

The effectiveness of surface finishing and polishing procedures on dental ceramics is commonly assessed through surface roughness measurements. For this reason, a contact-type profilometer—widely used in previous studies—was employed in the present investigation [19]. The results of our study have showed that the highest Ra and Rz values after grinding are observed in the ZLC group, followed by LDC and TrZ. Red-banded diamond burs with 50 μm grit are selected to simulate a clinical adjustment procedure. The effectiveness of grinding is influenced by the hardness difference between the abrasive instrument and the restorative material: diamond is the hardest known material, while the hardness of translucent zirconia (1485 HV) is considerably higher than of ZLC (700 HV) and LDC (580 HV) [20–22]. This likely explains why the lowest Ra and Rz values after grinding are observed in the zirconia group. The higher roughness seen in ZLC compared with LDC may also be attributed to differences in their microstructures.

Although the average Ra values of the ZLC-G group are slightly lower than those of ZLC-P; the difference is not statistically significant. In LDC, polishing has produced marginally lower Ra values than glazing, but again without statistical significance. Only in translucent zirconia did polishing result in significantly smoother surfaces compared with glazing. These findings suggest that chairside or laboratory polishing can serve as a viable alternative to glazing for achieving smooth surfaces in certain CAD/CAM ceramics. This interpretation is supported by recent reports indicating that appropriate polishing protocols can achieve surface smoothness comparable to-or even exceeding-that of glazing, particularly in zirconia [23].

In glass-ceramic materials, the superior smoothness achieved by glazing is attributed to the flow of low-fusing porcelain, which fills superficial irregularities during firing. In contrast, zirconia contains less than 1% SiO₂, which may explain why glazing is less effective than polishing. In this study, thermocycling increased surface roughness in all glazed groups, whereas polished groups showed more stable Ra values. Thermal stresses can induce microstructural deformation or microcrack formation in the glaze layer, leading to roughening over time. Additionally, the acidic pH of coffee and its potential to promote silica dissolution and alkali ion leaching may further compromise glaze integrity, contributing to the roughness increase observed after thermocycling [24].

Surface roughness of ceramic restorations is directly related to antagonist wear, highlighting the clinical importance of the finishing procedure [25–27]. Previous studies have consistently reported that monolithic zirconia and glass-ceramics exert greater abrasive effects on enamel compared with resin-matrix ceramics [28, 29]. Additionally, multiple investigations have shown that glaze layers tend to wear rapidly in the oral environment, exposing a rougher underlying surface. Our finding that polishing produced smoother and more stable surfaces on translucent zirconia aligns with recent evidence supporting the long-term reliability of mechanical polishing over glazing [30].

Regarding colour stability, the lowest ΔE values in our study were observed in TrZ-P, followed by LDC-G, TrZ-G, ZLC-G, LDC-P, with the highest recorded in ZLC-P. Although ZLC-P slightly exceeded the perceptibility threshold, all groups remained within clinically acceptable limits. Therefore, the observed colour differences should be interpreted cautiously, as statistical significance does not always correspond to clinically perceptible or unacceptable discoloration. Interpretation of discoloration was based on both statistical outcomes and established clinical threshold values [31, 32]. Glazing may seal microcracks created during grinding through thermal flow of the glassy matrix, reducing stain penetration and improving colour stability. The significantly higher colour change in ZLC-P may reflect microcracks that were not sealed by glazing, a mechanism supported by Riquieri et al. [33], who demonstrated that thermal treatment enhances the microstructure and mechanical properties of ZLC ceramics.

LDC exhibited higher translucency than ZLC, while translucent zirconia showed the lowest translucency values, consistent with the established optical behaviour of these materials [34]. The higher translucency of LDC can be attributed to its larger and more uniform crystal structure, whereas the higher crystal content of ZLC contributes to increased opacity. All groups demonstrated decreased TP values after thermocycling, with significant reductions in every group except LDC-P. Only LDC-G showed a translucency loss exceeding the perceptibility threshold, although it remained within clinically acceptable limits. Translucency reduction in zirconia following aging is often linked to tetragonal-to-monoclinic (t→m) phase transformation, which increases light scattering at grain boundaries [3, 35]. Our findings support this mechanism, as both zirconia groups exhibited notable TP decreases after aging.

A recent study by Walczak et al. [35] also reported decreased translucency in monolithic zirconia after autoclave aging, consistent with our observations. Differences in absolute TP values between studies may be attributed to variations in specimen thickness, aging protocols, and material formulations. Subaşı et al. [36] found no significant effect of coffee thermocycling on translucency for most materials at thicknesses of 0.7–1.0 mm, whereas our study detected significant TP loss in nearly all groups. Differences in specimen thickness, microstructure, and the glaze/polishing systems used may account for this discrepancy.

This in vitro study has inherent limitations in replicating the oral environment. Both surfaces of the disc specimens were exposed to the staining solution, only coffee was tested as a chromogenic agent, and dynamic occlusal forces were not simulated. SEM evaluation was qualitative, and post-aging SEM analysis was not conducted. These factors may limit the direct clinical applicability of the results. Nevertheless, the use of standardized grinding, glazing, polishing, and thermocycling protocols supports the reliability of the comparisons. Further clinical studies are required to validate these findings in vivo.

## Conclusion

Within the limitations of this in vitro study, the results indicate that ceramic material type, surface finishing protocol, and coffee thermocycling may be associated with variations in surface roughness, color stability, and translucency. Glazing was generally accompanied by more favorable color stability in glass-ceramics, whereas polishing was associated with lower surface roughness values in translucent zirconia. Thermocycling was also accompanied by a reduction in translucency; however, these changes remained within clinically acceptable ranges. Further clinical research is required to better understand these observations under intraoral conditions.

## Data Availability

The datasets used and/or analyzed during the current study are available from the corresponding author on reasonable request.

## Declarations

### Authors' information

This study is based on the thesis of Sena Kamacı Ergül.
